# Bronchitis

**Published:** 1891-05

**Authors:** 


### BRONCHITIS.

A good poultice for bronchitis is made with two tablespoonfuls of crushed linseed and one teaspoonful of dry mustard. This also it is better to mix in the invalid's room. Take a small basin, pour some boiling water into it to make it hot, then empty it, and pour a very little in quite boiling. Pick up the linseed with your left hand and scatter it into the basin ; with your right hand take a paper-knife and stir it as if you were making porridge, then add the dry mustard. Stir it until it becomes like a cake and does not stick to the dish. Then take a linen rag, the size you require, and put the linseed in the middle of it ; spread it perfectly smooth with a paper-knife ; cut away a very tiny bit of linseed all the way round, and turn the linen edge over it—this will prevent the linseed from falling. Feel the poultice in your hand to be sure it is not more than one-quarter inch thick, and lay it on the patient with the linseed next the skin. Be very careful that it covers the hollows at the top of the collar-bones, for there is the real danger of bronchitis, and not low down, as so many people imagine. A piece of flannel, just a size larger, should be laid over the poultice. When it is time to remove it, lift it gently as you would the mustard one, and wash the skin with warm water to cleanse it.

